# Tenth International Medical Congress

**Published:** 1890-05

**Authors:** 


THE COMMITTEE OF ORGANIZATION OF THE TENTH INTERNATIONAL MEDICAL CONGRESS.

Dr. Rudolf Virchow,

President.

Dr. E. von Bergmann, Dr. E. Leyden, Dr. W. Waldeyer,

Vice-Presidents.

Dr. O. Lassar,
Secretary-General.

---

### TENTH INTERNATIONAL MEDICAL CONGRESS.

TO BE HELD IN BERLIN, AUGUST 4TH TO 9TH.

The Committee of Organization of the Tenth International Medical Congress, R. Virchow, *President*; E. von Bergmann, E. Leydon, W. Waldeyer, *Vice-Presidents*; O. Lassar, *Secretary-General*, have appointed the undersigned members of an American Committee for the purpose of enlisting the sympathy and co-operation of the American profession.

We are assured that the medical men of our country will receive a hearty welcome in Berlin. The Congress promises to prove of inestimable value in its educational results, and in securing the ties of international professional brotherhood. It is most important that the American profession should participate both in its labors and its fruits.

Delegates of American Medical Societies and Institutions and individual members of the profession will be admitted on equal terms. The undersigned, therefore, beg to express their hope that a large number of the distinguished men of our country, will appreciate both the honor conferred by this cordial invitation and the opportunity afforded us to fitly represent American medicine.

The Congress will be held at Berlin, from the fourth to the ninth of August.

The arrangements in regard to a few general meetings and the main scientific work, which is delegated to the sections, are the same as in former sessions. A medico-scientific exhibition, the programme of which has been published a few weeks ago, is to form an ingredient part. It is to the latter that the Berlin Committee is very anxious that both the scientific and the secular press should be requested to give the greatest possible publicity.

The office of the Secretary-General is Karlstrasse 19, N. W., Berlin, Germany.

S. C. BUSEY, Washington, D. C.
WM. H. DRAPER, New York.
R. H. FITZ, Boston, Mass.
H. HUN, Albany, N. Y.
A. JACOBI, New York.
WM. T. LUSK, New York,
WM. OSLER, Boston, Mass.
WM. PEPPER, Philadelphia, Pa.
J. PEYRE PORCHER, Charleston, S. C.
J. STEWART, Montreal, Can.

